# Application of antimicrobial drugs in perioperative surgical incision

**DOI:** 10.1186/s12941-018-0254-0

**Published:** 2018-02-03

**Authors:** Xu Yang, Xurao Xiao, Lefeng Wang, Yue Ao, Yapeng Song, Huabing Wang, Huanan Wang

**Affiliations:** 1 College of Animal Sciences, Zhejiang University, Hangzhou, 310058 China; 2 Department of Hematology, Guangzhou Women and Children’s Medical Center, Guangzhou Medical University, Guangzhou, 510623 China; 3 College of Animal Science and Veterinary Medicine, Henan Agricultural University, Zhengzhou, 450002 China

**Keywords:** Surgical operation, Incision infection, Antibacterial drugs, Antibiotic

## Abstract

Infection in surgical incision often results in poor wound healing, and one of the main factors for wound infection is the use of antimicrobial agents. Rational use of antibiotics is one of the key factors to prevent incision infection in general surgery. The number of current clinical studies on antibiotic use before and during surgery is greater than that of systematic studies on antibiotic use after surgery. For the rational use of antibiotics and improvement of wound healing rate, researchers around the world have gradually focused on the use of antibiotics after surgery. Despite the familiarity on the concept of “rational use of antibiotics”, few clear and systematic studies were conducted to elucidate the effect of different antibiotics on wound healing. Therefore, this review focuses on the use of different types of antimicrobial agents in surgical wounds.

## Background

Surgical operation is a medical procedure performed by surgeons or professionals that use instruments to repair damage, arrest disease, or place an implant in a living body [1].

Incision infection that results in poor wound healing is a potential risk for many postoperative patients [2]. Surgical site infection (SSI) is a postoperative complication and an important part of nosocomial infection [2]. Factors affecting postoperative wound infection include operating room, type and duration of surgery [3, 4], surgical personnel, physiological states of patient [4, 5], and antimicrobial drug use [6]. Given that antibiotics are the most widely used antimicrobial agents, the rational use of this medicine is the key to prevent incision infection in surgical operations [7, 8].

The rational use of antibiotics indicates that antibiotics cannot be misused. Misuse of antibiotics includes use of the wrong antibiotics, in the wrong dose, for the wrong duration, and not in accordance with associated guidelines and principles [9]. Clinical studies on the preoperative and intraoperative use of antibiotics are more numerous than those about the postoperative use of antibiotics. Preoperative and intraoperative uses of antibiotics belong to prophylactic use, whereas postoperative use of antibiotic belongs to therapeutic use. In general surgery, the postoperative use of antibiotics is more ineffective than the prophylactic use of antibiotic [10, 11]. In fact, the prophylactic use of antibiotics cannot be conducted ideally, and the optimal time for prophylactic administration for clinical application is still controversial. Patients should use antibiotics rationally to restrict incision infection and increase wound healing rate. Many studies [6, 8, 9] involving clinical intervention and retrospective analysis highlighted the concept of rational use of antibiotics. However, no systematic report on the influence of different antibiotics on wound healing has been conducted.

This article focuses on various antimicrobials (especially on antibiotics) that are mainly used in surgical wounds to provide a reference for further understanding of the rational use and effect of antimicrobial treatment on surgical incision infection.

## Surgical operation

Surgery is an operation that destroys the integrity of the tissue or restores the integrity of damaged tissues. In early medicine, surgery was performed to cut, incise, and suture the body surface via simple manual methods. With the development of surgical technic and operating instruments (such as surgical knives, including electric, microwave, ultrasonic, and laser scalpel), operation can be performed at any site of the body. However, infection remains an issue for surgical operation. SSI is a main obstructive factor restraining the success of surgical treatment.

### Surgical site infection

SSI is a postoperative complication and one of the most important healthcare-associated infections [12]. SSI includes infections in superficial incision, deep incision, and organ/tissue space (such as encephalopyosis, and peritonitis) [13]. Superficial incision includes incisions in the skin and subcutaneous tissue, deep incision refers to incisions in deep soft tissues (such as fascia and muscle layer), and organ/tissue space refers to any anatomical structure that has been opened or treated by surgery. The diagnostic criterion for SSI is that the infection occurs within 30 days after operation or within 1 year after implantation [13], that is, the presence of a purulent exudate within the said period can be identified as an SSI. The presence of fever, local tenderness or pain, incision swelling, and other symptoms should prompt diagnosis by clinician after microbial cultivation.

SSI related deaths account for more than one-third of postoperative mortalities worldwide [14]. The incidences of SSI in developed countries as USA (1.9%) [12], France (1.0%) [15] and Italy (2.6%) [16], are lower than those in developing nations including Turkey (4.1%) [17], China (4.5%) [18] and India (5.0%) [19]. The risk factors for SSI identified include the length of preoperative hospital stay, wound class and duration of surgery, which influence the breeding and proliferation of pathogens [20]. Among the pathogens that cause SSI, 48% of which are Gram-negative, 40.8% are Gram-positive, and 11.2% are fungi [21]. In China, *Escherichia coli* (25.9%), *Staphylococcus aureus* (14.3%), and *Pseudomonas aeruginosa* (11.9%) are the three dominate pathogens [22]. In the surgical incisions or wounds, the proliferation of pathogens usually results in postoperative infection. The rational use of antimicrobial agents can effectively reduce the occurrence of SSI [23].

### Surgical incision

The occurrence of SSI is related to the contamination of trauma during the operation. Surgical incisions were previously divided into the following three categories [24]: type I, clean incision; type II, possible contamination incision; and type III, contamination incision. According to healing and infection, an incision was classified into grades A, B, or C. However, this classification method is not perfect in practice. Incision cases are generally divided into four groups (Table 1) [25] to better assess the incision contamination. An in-depth understanding of the standard classification of surgical incisions is beneficial for the monitoring and prevention of SSI and provides a strong theoretical basis for the application of antimicrobial agents.

**Table 1:** Incision classification

Classification	Definition
Class I	
Clean incisions	An uninfected surgical incision in which no inflammation is encountered and the respiratory, alimentary, genital, or uninfected urinary tracts are not entered. Surgical wound incisions that are made after nonpenetrating trauma should be included in this category if they meet the criteria
Class II	
Clean-contaminated incisions	A surgical incision in which the respiratory, alimentary, genital, or urinary tracts are entered under controlled conditions and without unusual contamination. Specifically, surgical procedures involving the biliary tract, appendix, vagina, and oropharynx are included in this category, provided no evidence of infection is encountered
Class III	
Contaminated incisions	Open, fresh, accidental incisions. In addition, surgical procedures in which a major break in sterile technique occurs (e.g., open cardiac massage) or there is gross spillage from the gastrointestinal tract and incisions in which acute, nonpurulent inflammation is encountered are included in this category
Class IV	
Dirty/infected incisions	Old traumatic incisions with retained or devitalized tissue and those that involve existing clinical infection or perforated viscera

## Antimicrobial agents

Antimicrobial agents, including various antibiotics and synthetic antibiotics that have bactericidal or bacteriostatic effects, are drugs that kill or inhibit the effects of bacteria [26]. In 1999, four classification methods were applied based on chemical structure, action mode, antimicrobial spectrum, and action mechanism of antimicrobial agents [27]. In 2004, Zhao [28] established a new interpretation for the classification of antimicrobial agents in the book Manual of Modern Pharmic Terms. Based on their biological activity, antibacterial drugs were divided into anti-Gram-positive bacteria, anti-Gram-negative bacteria, broad-spectrum antibiotics, anti-anaerobia drugs, anti-*Mycobacterium tuberculosis*, and beta-lactamase inhibitors. Based on their action mechanisms, antibacterial drugs include those interfering with cell wall synthesis, inhibiting protein synthesis, interfering with nucleic acid synthesis, inhibiting metabolic pathways, and those disrupting bacterial membrane structures [29]. Based on their chemical structure, antibacterial drugs are classified as beta-lactams (e.g., penicillin), aminoglycosides (e.g., streptomycin), tetracycline (e.g., tetracycline), macrolides (e.g., erythromycin), sulfonamides (e.g., sulfadiazine), quinolones (e.g., norfloxacin), anti-mycobacterium tuberculosis (e.g., isoniazid), and other antibacterial drugs (such as clindamycin, vancomycin, fosfomycin, and bacitracin). In 2014, Zhuo and Zhong [30] classified the main antimicrobial agents as beta-lactams, macrolides, aminoglycosides, and quinolones.

### Antimicrobial agents for external use

Some common antibacterial agents for external use are listed in Table 2 [31]. Application of these agents in open surgical incision or trauma treatment results in quick-acting and broad-spectrum antibacterial activity. Considering that these antibacterial agents cannot easily reach the site of infection through oral administration or intravenous injection, their external use is optimal. In addition, different antimicrobial agents have different antibacterial mechanisms and effectiveness on the same pathogen. An antibacterial agent has different antibacterial mechanisms and inhibition ranges for different pathogens to impede the development of drug-resistant strains.

**Table 2:** Antiseptic agents applied to open surgical incisions and used to prevent or manage post-operative surgical site infection

Antiseptic choice	Comments
Acetic acid solution	Diluted solution (0.25%) has been used in incisions with no evidence to support use. Higher concentrations may cause tissue injury. The solution has been used for otitis external management and bladder irrigation
Alcohols	Isopropyl alcohol with rapid bactericidal efficacy is most commonly used for topical skin application. Not recommended for application in open incisions, because it desiccates open incisions and is tissue toxic
Boric acid	It is used as eye irrigation, and not recommended for application in open incisions
Chlorhexidine	It is the most common antiseptic in skin preparation, and generally not recommended in open incisions at the conventional concentration (2–4%)
Chlorine compounds	Buffered sodium hypochlorite solution at a concentration of 0.25–0.5%, leading to oxidative injury of bacterial membranes and enzymes
Hydrogen peroxide	It is a common household remedy for cuts and bruises, and is generally tissue-toxic for use in open incisions
Iodine compounds	Tincture of iodine has almost disappeared for any application in operations. Iodophors, which are less toxic, have been used clinically for topical application in open incisions but have not been subject to evaluation for efficacy
Silver compounds	Silver nitrate and silver sulfadiazine have been widely used in burns and selectively in open infected incisions of soft tissue. Various sustained-release topical preparations are available but are not used commonly for prevention in open incisions
Triclocarban	A common antiseptic in commercial soaps; there is no data to evaluate its use in open incisions
Triclosan	Common antiseptic in cosmetics; used in the coating for selected surgical sutures. No data on use for open incisions

However, the side effects of antimicrobial agents limit their application in acute surgical incision treatment. Many antimicrobial agents have concentration-dependent tissue toxicity, and some of their components enhance local toxicity [32, 33]. Moreover, antimicrobial agents may also interfere with wound healing [34–36].

### Antibiotics

Antibiotics are secondary metabolites and derivatives with physiological activity produced by microorganisms, including secondary metabolites produced by bacteria, molds, and other microorganisms, as well as synthetic analogues [37]. Antibiotics at low concentrations could selectively inhibit or interfere with normal life activities of other microbes. Antibiotics should be utilized rationally because the abuse of antibiotics causes serious consequences. In 2002, Zhang [38] introduced the rational use of antibiotics. In the Journal of Modern Laboratory Medicine issued in 2007, several scholars mentioned bacterial resistance and the necessity of rational use of antibiotics [39]. The overuse of antibiotics can lead to superbugs—a progressively escalating challenge [40, 41]. In 2010, Kumarasamy et al. [42] firstly reported the presence of NDM-1 superbugs, a drug resistant bacterium with a high replication capacity and high speed of transmission. The abusive use of antibiotics may lead to the development of superbugs, and bacteria can develop acquired resistance to antibiotics that they have encountered [43, 44]. Many powerful superbugs develop with the continual abuse of powerful new antibiotics [45]. With the current research on clinical intervention and retrospective analysis, the rational use of antibiotics has received increasing attention. World Health Organization (WHO) has highlighted the importance of rational use of antibiotics and warned that drug-resistance caused by irrational use of antibiotics will result in frequent infections and even a small injury may result in death [46]. The rational use of antibiotics implies no overtime, no excessive use, symptomatic use, and the use of antibiotics in strict accordance with the standards. The rationality of the use of antibiotics is affected by various factors, including the type of surgical incision, the type of antibiotics and antibiotic combinations, and the time of administration. According to the time of administration, antibiotics can be classified into preventive medicine and therapeutic medicine.

#### Prophylactic medication

Prophylactic administration of antibiotics refers to the administration of antibiotics prior to the occurrence of contamination and the release of antibiotics to the site of surgery to ensure sufficient concentration of antibiotics in target tissues [47, 48]. Antibiotics are usually given before and during surgery. In 2000, Xi showed that prophylactic antibiotics should reach the tissues before bacterial contamination occurs; when the tissue contamination exceeds 4 h, the antibiotics lose their preventive effect [49]. In 2007, a prospective study of incisional infection showed that prophylactic medication administered at 0.5–1 h before surgery leads to a lower rate of incision infection than those administered 2 h preoperative [50]. Reasonable prophylactic use of antibiotics before surgery can effectively reduce the rate of postoperative infection [51]. Reasonable time of prophylactic administration greatly reduces postoperative infections. However, the prophylactic application of antibiotics is still far from perfect in general surgery [52, 53]. Strengthening the management and rational use of antibiotics is an urgent goal. In 2013, a guideline was issued for antimicrobial prophylaxis in surgery, which aims to contribute to the reasonable use of antibiotics [54]. In 2016, the WHO launched the first global guideline on the use of antibiotic prophylaxis to prevent SSI [55]. The guideline will have applicability in all countries and be especially welcomed in developing countries [56, 57]. The selection and application of prophylactic antibiotics should be conducted according to medical characteristics including the type of surgical incision, the possibility of infection, the type of potential pathogens and the prophylactic effect of antibiotics [58]. The best timing for the surgical antibiotics administration depends on the pharmacokinetics of each antibiotic. Guidelines provide divergent duration comprised from 30 to 60 min before incision [59, 60]; while exceptions including fluoroquinolones and vancomycin are recommended to be administrated within 120 min [55, 59]. A single administration is recommended in most cases and the favorite route of administration is intravenous [58], except for specific procedures as prostate surgery [61]. Prophylactic antibiotics are generally not recommended in type I incisions, except occasions below: (1) operation which last long and involve more tissues, leading to high risk of infection; (2) operation which involve the vital organs including heart, lung and brain; (3) operation which involve foreign body implantation; (4) patients with high risk factors of infection, such as malnutrition, immune dysfunction and diabetes [62]. Any drug used for type IV incisions doesn’t belong to prophylactic medicine, because the infections occur before surgery. Therefore, prophylactic antibiotics are normally used for type II and III incisions. The choices of prophylactic antibiotics for perioperative surgical incisions (type II and III) are listed in Table 3. Patients with prophylactic antibiotic use had lower rates of postoperative infection [63].

**Table 3:** The choices of prophylactic antibiotics for perioperative surgical incision

Operation name	Incision type	Potential pathogens	Antimicrobial agents
Ophthalmic surgery	I, II	*Staphylococcus aureus* (*S. aureus*), coagulase negative Staphylococci (CNS)	Tobramycin or levofloxacin in topical application
Amputation	I, II	*S. aureus*, CNS, Streptococcus (Strep), Gram-negative bacilli (GNB), Anaerobic bacteria (AB)	(First or second generation) cephalosporin (Cepha) ± metronidazole (MNZ)
Cerebral surgery (through the nasal sinuses, nasal cavities and oropharynx)	II	*S. aureus*, Strep, oropharyngeal AB (e.g., Peptostreptococcus)	Cepha ± MNZ, or clindamycin + gentamicin (C + G)
Head and neck surgery (through the oropharynx mucosa)	II	*S. aureus*, Strep, oropharyngeal AB (e.g., peptostreptococcus)	Cepha ± MNZ, or C + G
Otorhinolaryngologic surgery	II	*S. aureus*, CNS	Cepha ± MNZ
Thoracic surgery	II	*S. aureus*, CNS, Strep pneumoniae, GNB	Cepha ± MNZ
Urology surgery (entering the urinary tract vaginal)	II	GNB	Cepha or fluoroquinolones
Urology surgery (involving intestinal tract)	II	GNB, AB	Cepha or aminoglycosides (AG) + MNZ
Urology surgery (with prosthesis implantation)	II	Staphylococcus, GNB	Cepha + AG, or vancomycin
Hysterectomy	II	GNB, Enterococcus, Group B Streptococci (GBS), AB	Cepha (plus MNZ in vaginal surgery), or cephamycin
Laparoscopic myomectomy (using uterine manipulator)	II	GNB, Enterococcus, GBS, AB	Cepha ± MNZ, or cephamycin
Premature rupture of amniotic membrane or cesarean section	II	GNB, *Enterococcus* spp., GBS, AB	Cepha ± MNZ
Artificial abortion—curettage odinopoeia	II	GNB, *Enterococcus* spp., GBS, AB	Cepha ± MNZ, or doxycycline
Flap transfer or skin grafting	II	*S. aureus*, CNS, Strep, GNB	Cepha
Implantation of external fixator	II	*Staphylococcus aureus*, CNS, Strep	Cepha
Open fracture internal fixation	II	*Staphylococcus aureus*, CNS, Strep, GNB, anaerobic bacteria	Cepha ± MNZ
Hepatic, biliary, and pancreatic surgery	II, III	CNS, AB	Cepha or ceftriaxone ± MNZ, or cephamycin
Stomach, duodenum, small intestine surgery	II, III	CNS, Strep, oropharyngeal AB	Cepha or cephamycin
Colon, rectum, appendectomy	II, III	CNS, AB	Cepha or ceftriaxone ± MNZ, or cephamycin
Repair of perineal laceration	II, III	GNB, Enterococcus, GBS, AB	Cepha or ceftriaxone ± MNZ

± That two or more drugs can used in combination or not in combination

#### Therapeutic medication

Therapeutic use of antibiotics indicates that antibiotics are used and released to the surgical site after contamination to exert their antibacterial effect. The prophylactic application of antibiotics leads to a reduced risk of postoperative infection. However, preventive administration can only reduce and not completely eradicate the possibility of postoperative infection [64–66]. Therefore, therapeutic drugs are still necessary sometimes. Also, therapeutic drugs are required for cases with preoperative infection, such as type IV incisions. For surgical infection, the proper selection of antibiotics and effective treatment of are based on the correct diagnosis, the location of the infection, the patient’s condition, the pharmacokinetics and pharmacodynamics of antimicrobials, and so on [8]. When antibiotics are selected to treat surgical infection, pathogens should be identified first. Normally, pathogens can be identified by culturing infected tissues, secretions, or blood samples. Prior to the test results, an infection with a definite diagnosis may be treated according to clinical experience. For example, *Pseudomonas aeruginosa* produces blue-green secretions and can be treated with gentamicin or polymyxin [67]. After pathogens are identified, clinicians should choose antibiotics according to their properties including spectrum, pharmacokinetics, pharmacodynamics and clinical efficacy. For instance, urinary tract infection can treated with aminoglycosides which are mainly excreted through the kidneys. However, aminoglycosides aren’t the best choice because of their high toxicity [68, 69]. We can choose less toxic drugs, such as sulfonamides, furans and fluoroquinolones, which can also keep effective concentrations in the urethra. The patient’s condition is also an important consideration in the choice of antibiotics. For children, the dosage of antibiotics should be reduced appropriately; and for older people, who metabolize and excrete antibiotics at a slower pace, there should be longer intervals between the doses of antibiotics [70]. In a word, the rational use of drugs is the key to the prevention and treatment of surgical infection.

## Summary

Incisions are mostly performed in surgical operations, and surgical incision infections can cause poor surgical wound healing. Postoperative incision infection is often called surgical site infection, one of the complications after the operation and an important part of hospital infection. Antimicrobial agents are a factor affecting the prevention and treatment of postoperative infections. Therefore, studies on the range of application, incompatibility, method of administration, treatment time, adverse reactions, and other related characteristics of various antibiotics have been initiated. As a common antibacterial drug, antibiotics have been widely used by medical personnel. However, during the use of antibiotics, some adverse effects have emerged because of antibiotics abuse. In addition, the characteristics of certain antibiotics have not been clearly defined. Postoperative antibiotics used in clinics have gradually become the research focus worldwide to achieve the rational use of antibiotics and improve the wound healing rate. Exploring the types of antibiotics used in clinical practice will help medical staff to prevent and treat surgical infection, thereby indicating its far-reaching scientific and clinical significance.

## Abbreviation

SSI: surgical site infection

## References

[1] Bass BL, Garbey M (2014). A road map for computational surgery: challenges and opportunities. J Comput Surg..

[2] Bratzler DW, Houck PM (2004). Antimicrobial prophylaxis for surgery: an advisory statement from the national surgical infection prevention project. Clin Infect Dis.

[3] Medeiros AC, Aires-Neto T, Azevedo GD, Vilar MJ, Pinheiro LA, Brandao-Neto J (2005). Surgical site infection in a university hospital in northeast Brazil. Braz J Infect Dis..

[4] Kaya E, Yetim I, Dervisoglu A, Sunbul M, Bek Y (2006). Risk factors for and effect of a 1-year surveillance program on surgical site infection at a university hospital in Turkey. Surg Infect.

[5] Pravinkumar E (2003). Obesity in general elective surgery. Lancet..

[6] de Jonge SW, Gans SL, Atema JJ, Solomkin JS, Dellinger PE, Boermeester MA (2017). Timing of preoperative antibiotic prophylaxis in 54,552 patients and the risk of surgical site infection: a systematic review and meta-analysis. Medicine.

[7] Zhou C, Chen X, Wu L, Qu J (2016). Distribution of drug-resistant bacteria and rational use of clinical antimicrobial agents. Exp Ther Med..

[8] Lambrini K, Kotsiftopoulos CH, Papageorgiou M, Iliadis CH, Monios A (2017). The rational use of antibiotics medicine. J Healthc Commun..

[9] Saini N, Saini V, Mehta PW (2014). Misuse of antibiotics: a potential threat. IOSR J Dent Med Sci..

[10] Young RF, Lawner PM (1987). Perioperative antibiotic prophylaxis for prevention of postoperative neurosurgical infections. A randomized clinical trial. J Neurosurg..

[11] Dellinger EP (2007). Prophylactic antibiotics: administration and timing before operation are more important than administration after operation. Clin Infect Dis.

[12] Mu Y, Edwards JR, Horan TC, Berrios-Torres SI, Fridkin SK (2011). Improving risk-adjusted measures of surgical site infection for the national healthcare safety network. Infect Control Hosp Epidemiol.

[13] Mangram AJ, Horan TC, Pearson ML, Silver LC, Jarvis WR (1999). Guideline for prevention of surgical site infection, 1999. Hospital infection control practices advisory committee. Infect Control Hosp Epidemiol..

[14] Awad SS (2012). Adherence to surgical care improvement project measures and post-operative surgical site infections. Surg Infect.

[15] Saunders L, Perennec-Olivier M, Jarno P, L’Heriteau F, Venier AG, Simon L (2014). Improving prediction of surgical site infection risk with multilevel modeling. PLoS ONE.

[16] Marchi M, Pan A, Gagliotti C, Morsillo F, Parenti M, Resi D (2014). The Italian national surgical site infection surveillance programme and its positive impact, 2009 to 2011. Euro Surveill..

[17] Isik O, Kaya E, Dundar HZ, Sarkut P (2015). Surgical site infection: re-assessment of the risk factors. Chirurgia (Bucur)..

[18] Fan Y, Wei Z, Wang W, Tan L, Jiang H, Tian L (2014). The incidence and distribution of surgical site infection in mainland China: a meta-analysis of 84 prospective observational studies. Sci Rep.

[19] Pathak A, Saliba EA, Sharma S, Mahadik VK, Shah H, Lundborg CS (2014). Incidence and factors associated with surgical site infections in a teaching hospital in Ujjain, India. Am J Infect Control..

[20] Carvalho R, Campos CC, Franco L, Rocha AM, Ercole FF (2017). Incidence and risk factors for surgical site infection in general surgeries. Rev Lat Am Enfermagem..

[21] Hendren S, Fritze D, Banerjee M, Kubus J, Cleary RK, Englesbe MJ (2013). Antibiotic choice is independently associated with risk of surgical site infection after colectomy: a population-based cohort study. Ann Surg.

[22] Wen X, Ren N, Wu A (2012). Distribution of pathogens and antimicrobial resistance: an analysis of China healthcare-associated infection cross-sectional survey in 2010. China J Infect Cont..

[23] Alp E, Elmali F, Ersoy S, Kucuk C, Doganay M (2014). Incidence and risk factors of surgical site infection in general surgery in a developing country. Surg Today.

[24] Group CC (2003). Guideline for prophylactic and therapeutic use of antimicrobial agents in surgical patients (working draft). Chin J Pract Surg..

[25] Giarrizzo-Wilson S, Maxwell-Downing D, Chard R (2008). Clinical issues. AORN J.

[26] Saga T, Yamaguchi K (2009). History of antimicrobial agents and resistant bacteria. Jpn Med Assoc J..

[27] Bin X (1999). How are antimicrobial agents classified?. Chin J Med..

[28] Kejian Z (2004). Manual of modern pharmic terms.

[29] Tenover FC (2006). Mechanisms of antimicrobial resistance in bacteria. Am J Med..

[30] Chao Z, Nanshan Z (2014). Antimicrobial agents and their rational clinical applications. Chin J Joint Surg.

[31] Fry DE (2016). Topical antimicrobials and the open surgical wound. Surg Infect.

[32] Craig WA, Kunin CM (1976). Significance of serum protein and tissue binding of antimicrobial agents. Annu Rev Med.

[33] Sy SK, Malmberg R, Matsushima A, Asin-Prieto E, Rosenkranz B, Cotton MF (2015). Effect of reducing the paediatric stavudine dose by half: a physiologically-based pharmacokinetic model. Int J Antimicrob Agents.

[34] Barnard RD, Gillman J (1952). Delayed wound healing: a possible complication of Streptomyces-derived antibiotic administration. Urol Cutan Rev.

[35] Geronemus RG, Mertz PM, Eaglstein WH (1979). Wound healing. The effects of topical antimicrobial agents. Arch Dermatol.

[36] Praessler J, Bauer A, Elsner P, Kaatz M (2005). Contact dermatitis to quinoline, corticosteroids and antibiotics after short time treatment of delayed wound healing following malignant melanoma excision in a young woman. Skin Res Technol..

[37] Pool C, Kass J, Spivack J, Nahumi N, Khan M, Babus L (2016). Increased surgical site infection rates following clindamycin use in head and neck free tissue transfer. Otolaryngol Head Neck Surg..

[38] Yongxin Z (2002). Rational use of antibacterial agents. Chin J Pediatr..

[39] Qiongying Q, Jilong L, Jianling L (2007). Surveillance of bacterial resistance and rational use of antibiotics in clinical practice. J Mod Lab Med..

[40] Morris A, Kellner JD, Low DE (1998). The superbugs: evolution, dissemination and fitness. Curr Opin Microbiol.

[41] Skariyachan S, Mahajanakatti AB, Sharma N, Karanth S, Rao S, Rajeswari N (2012). Structure based virtual screening of novel inhibitors against multidrug resistant superbugs. Bioinformation.

[42] Kumarasamy KK, Toleman MA, Walsh TR, Bagaria J, Butt F, Balakrishnan R (2010). Emergence of a new antibiotic resistance mechanism in India, Pakistan, and the UK: a molecular, biological, and epidemiological study. Lancet Infect Dis..

[43] Carmichael G (2004). ESBLs: the next challenge in infection control. Lancet Infect Dis..

[44] Lee HH, Molla MN, Cantor CR, Collins JJ (2010). Bacterial charity work leads to population-wide resistance. Nature.

[45] Cheng G, Dai M, Ahmed S, Hao H, Wang X, Yuan Z (2016). Antimicrobial drugs in fighting against antimicrobial resistance. Front Microbiol..

[46] Zaman SB, Hussain MA, Nye R, Mehta V, Mamun KT, Hossain N (2017). A review on antibiotic resistance: alarm bells are ringing. Cureus..

[47] Ludwig KA, Carlson MA, Condon RE (1993). Prophylactic antibiotics in surgery. Annu Rev Med.

[48] Widdison AL, Pope NR, Brown EM (1993). Survey of guidelines for antimicrobial prophylaxis in surgery. J Hosp Infect.

[49] Yiqun X, Hengjin D (2000). Antibiotic prophylaxis and related economic evaluation in surgical operations. Shanghai Med J..

[50] Guangxia M (2006). Prospective monitoring and intervention of surgical incision infection. J Qilu Nurs..

[51] Shizhou Z, Jianxiong Y, Xiaoyun X, Yingquan X, Xiangtao L (2012). Study on intervention of the rational application of antibiotics before surgical operation. Qilu Pharm Aff..

[52] Shinagawa N (2004). Antimicrobial prophylaxis in surgery. Jpn J Antibiot.

[53] Bowater RJ, Stirling SA, Lilford RJ (2009). Is antibiotic prophylaxis in surgery a generally effective intervention? Testing a generic hypothesis over a set of meta-analyses. Ann Surg.

[54] Bratzler DW, Dellinger EP, Olsen KM, Perl TM, Auwaerter PG, Bolon MK (2013). Clinical practice guidelines for antimicrobial prophylaxis in surgery. Surg Infect.

[55] Global Guidelines for the Prevention of Surgical Site (2016). Infection.

[56] Sway A, Solomkin JS, Pittet D, Kilpatrick C (2018). Methodology and background for the World Health Organization global guidelines on the prevention of surgical site infection. Surg Infect..

[57] Leaper DJ, Edmiston CE (2017). World Health Organization: global guidelines for the prevention of surgical site infection. J Hosp Infect.

[58] Duclos G, Zieleskiewicz L, Leone M (2017). Antimicrobial prophylaxis is critical for preventing surgical site infection. J Thorac Dis..

[59] Allegranzi B, Bischoff P, de Jonge S, Kubilay NZ, Zayed B, Gomes SM (2016). New WHO recommendations on preoperative measures for surgical site infection prevention: an evidence-based global perspective. Lancet Infect Dis..

[60] Berrios-Torres SI, Umscheid CA, Bratzler DW, Leas B, Stone EC, Kelz RR (2017). Centers for disease control and prevention guideline for the prevention of surgical site infection, 2017. JAMA Surg..

[61] Sampol-Manos E, Leone M, Karouia D, Savelli V, Ragni E, Rossi D (2006). Prophylaxis with ciprofloxacin for open prostatectomy: comparison of tissue penetration with two oral doses. J Chemother.

[62] National Health and Family Planning Commission of the People’s Republic of China (2016). Guiding principles for clinical application of antibiotics.

[63] Butterworth P, Terrill A, Barwick A, Hermann R (2017). The use of prophylactic antibiotics in podiatric foot and ankle surgery. Infect Dis Health.

[64] Najjar PA, Smink DS (2015). Prophylactic antibiotics and prevention of surgical site infections. Surg Clin North Am.

[65] Lo CW, Yang SS, Hsieh CH, Chang SJ (2015). Effectiveness of prophylactic antibiotics against post-ureteroscopic lithotripsy infections: systematic review and meta-analysis. Surg Infect.

[66] Rosen SA, Getz AE, Kingdom T, Youssef AS, Ramakrishnan VR (2016). Systematic review of the effectiveness of perioperative prophylactic antibiotics for skull base surgeries. Am J Rhinol Allergy..

[67] Zhang JF, Zhu HY, Sun YW, Liu W, Huo YM, Liu DJ (2015). *Pseudomonas aeruginosa* infection after pancreatoduodenectomy: risk factors and clinic impacts. Surg Infect.

[68] Hong S, Harris KA, Fanning KD, Sarachan KL, Frohlich KM, Agris PF (2015). Evidence that antibiotics bind to human mitochondrial ribosomal RNA has implications for aminoglycoside toxicity. J Biol Chem.

[69] Jackson J, Chen C, Buising K (2013). Aminoglycosides: how should we use them in the 21st century?. Curr Opin Infect Dis..

[70] Jetha S (2015). Polypharmacy, the elderly, and deprescribing. Consult Pharm..

